# Effective Control of Bioelectricity Generation from a Microbial Fuel Cell by Logical Combinations of pH and Temperature

**DOI:** 10.1155/2014/186016

**Published:** 2014-03-11

**Authors:** Jiahuan Tang, Ting Liu, Yong Yuan, Li Zhuang

**Affiliations:** ^1^Guangzhou Institute of Geochemistry, Chinese Academy of Sciences, Guangzhou 510640, China; ^2^Guangdong Institute of Eco-Environmental and Soil Sciences, Guangzhou 510650, China; ^3^College of Bioscience and Biotechnology, Hunan Agricultural University, Changsha 410128, China

## Abstract

In this study, a microbial fuel cell (MFC) with switchable power release is designed, which can be logically controlled by combinations of the most physiologically important parameters such as “temperature” and “pH.” Changes in voltage output in response to temperature and pH changes were significant in which voltage output decreased sharply when temperature was lowered from 30°C to 10°C or pH was decreased from 7.0 to 5.0. The switchability of the MFC comes from the microbial anode whose activity is affected by the combined medium temperature and pH. Changes in temperature and pH cause reversible activation-inactivation of the bioanode, thus affecting the activity of the entire MFC. With temperature and pH as input signals, an AND logic operation is constructed for the MFC whose power density is controlled. The developed system has the potential to meet the requirement of power supplies producing electrical power on-demand for self-powered biosensors or biomedical devices.

## 1. Introduction

Microbial fuel cells (MFCs) are energy-producing devices that can directly generate electric energy from the oxidation of various organic matters with the aid of microbes. Since MFCs are capable of generating electricity in an environmentally friendly manner, they have attracted a great deal of research attention over the past decades [1–3]. One of the potentially important applications of MFCs is powering sensors or biomedical devices. Tender et al. [4] and Zhang et al. [5] succeeded in powering temperature sensors using MFCs in natural environments. Siu et al. proposed that MFCs were capable of powering biological microelectromechanical systems (bioMEMS) [6]. Recently, major research concerns have been raised about increasing energy output of MFCs as power supplies. In the case of powering sensors or biomedical devices, switchable or tunable electrical power release would be a great advantage for their application, given their adaptive behavior and power production on demand.

Enzyme-based biofuel cells with self-regulating power release have been intensively studied using switchable biocatalytic electrodes controlled by physical or biochemical signals [7]. In such devices, the biocatalytic electrodes were usually modified with signal-responsive materials that were sensitive to triggering actions to make the electrodes electrochemically active or inactive. For instance, Katz and Willner reported that the power production of a biofuel cell was switched ON and OFF by integrating a biocatalyst with a copper polyacrylic acid matrix of controllable conductivity properties [8]. Amir et al. regulated the power release of a biofuel cell using a pH-sensitive redox polymer [9]. The polymer allowed switching between redox active and inactive states by pH-induced swelling and shrinking processes, respectively. Other biofuel cell systems with controllable power release were also realized by being integrated with enzyme- or immune-based systems, enabling the switchable and tunable functions of the biofuel cells [10, 11]. Although promising progress has been achieved for enzyme-based biofuel cells, their long-term stability and scalability need to be improved, and the complicated manufacturing process of enzyme electrodes severely limit their practical application. MFCs are promising alternatives to enzyme-biofuel cells due to the use of whole bacterial cells which are stable for a long time, scalable, self-attachable on the electrodes as catalysts. Microbial electrodes or assembled MFCs are capable of switching the electron transfer process or self-regulating power release as well. Yuan et al. developed a switchable microbial electrode with electrical signal output by controlling the presence of acetate and oxygen [12]. For the first time, Li et al. integrated an MFC with an AND logic gate to self-regulate power release [13]. However, a* Pseudomonas aeruginosa* lasI/rhlI double mutant was employed as the biocatalyst and two quorum-sensing signaling molecules were used as input signals in their study, involving rather complex genetic operations and slow switching speed.

In this study, we demonstrated a simple method to regulate the electricity generation from an MFC by controlling the most important physiological parameters (pH and temperature), where a sharp thermal and pH response of the microbial anode of the MFC has been logically designed. The bioanode could switch between electrochemically active and inactive states in response to the operation environment, resulting in the “smart” bioelectricity generation from the assembled MFC. The present paper extends the fundamental research activity in bioelectricity generation of MFCs with switchable or tunable functions.

## 2. Materials and Methods

### 2.1. MFC Construction and Startup

MFCs with an inner volume of 12 mL were constructed as previously reported with minor modification on the anodes [14]. A cylindrical MFC chamber with a length of 1.7 cm and a diameter of 3.0 cm in the cathode side and 1.8 cm in the anode side was made of plexiglass, resulting in a surface area of  7.0 cm^2^ for the cathode and 2.5 cm^2^ for the anode. The cathode was prepared with a 30% wet-proof carbon cloth (type B, E-TEK, USA) with four layers of polytetrafluoroethylene (PTFE) (PTFE30, DuPont, USA) coating. Pt/C (20% Pt, E-TEK, USA) was used as the cathode catalyst with a Pt loading of 0.5 mg cm^−2^. Carbon cloth (type A, E-TEK, USA) was used as the anode electrode. Reactors were inoculated with 2 mL activated sludge (Liede Wastewater Treatment Plant, Guangzhou, China) in 10 mL sodium acetate (1000 mg L^−1^) culture medium. Besides sodium acetate, the culture medium contained KH_2_PO_4_ (13.6 g L^−1^), NaOH (2.32 g L^−1^), NH_4_Cl (0.31 g L^−1^), NaCl (1.0 g L^−1^), a vitamin stock solution (12.5 mL L^−1^), and a mineral stock solution (12.5 mL L^−1^). Power density curves were obtained by changing the circuit resistor from 50 Ω to 5000 Ω. All tests were conducted in duplicate, and mean values were presented.

### 2.2. UV-Vis Spectroscopy

Prior to spectroscopy measurements, pure* Geobacter* strain was cultured and harvested.* Geobacter sulfurreducens* strain PCA (ATCC 51573) was cultured as previously reported at 30°C using a vitamin-free anaerobic medium [15]. Acetate was provided as an electron donor at 30 mM and 40 mM fumarate as electron acceptor. UV-Vis spectra of intact cells of PCA were recorded in diffused transmission mode with bacterial cells suspended in a bicarbonate buffer [16]. The cell suspension was injected into a cuvette, and it was mounted in front of an integrating sphere to measure the diffuse transmission light. Full reduction of the cells was achieved by adding sodium dithionite (25 mM) to the cell suspension and oxidation of the whole cell was obtained by purging oxygen into the cell suspension for 10 min before measuring.

### 2.3. Electrochemical Measurements

Electrochemical characterization of the mixed-culture anode was carried out with cyclic voltammetry (CV) using a CHI660D workstation (Shanghai CH Instrument Company, China) with a three-electrode system, where the biofilm-attached anode served as the working electrode, saturated calomel electrode (SCE) as the reference electrode, and the cathode as the counter electrode. A phosphate buffer (0.05 M; no culture medium) was used as the electrolyte under no-turnover conditions. CV under turnover conditions was measured when the voltage output of the MFC at 1000 Ω was maximized and stabilized.

### 2.4. Scanning Electron Microscopy

Prior to scanning electron microscopy (SEM) measurements, the mixed-culture biofilm-attached electrode was first fixed in a 2.5% glutaraldehyde solution for 1 h, then in a series of ethanol dehydration solution (i.e., 25%, 50%, 75%, and 100% v/v EtOH; 0.5 h each treatment), and after that, dried at the CO_2_-critical point for 3 h. The resultant specimen was coated with gold using a coating device (Emitech K550X; UK) and observed under a SEM (JEOL, JSM-6330F; Japan) at 20 kV.

## 3. Results and Discussion

### 3.1. Responses of Voltage Output to pH and Temperature Variations

**Figure 1:**
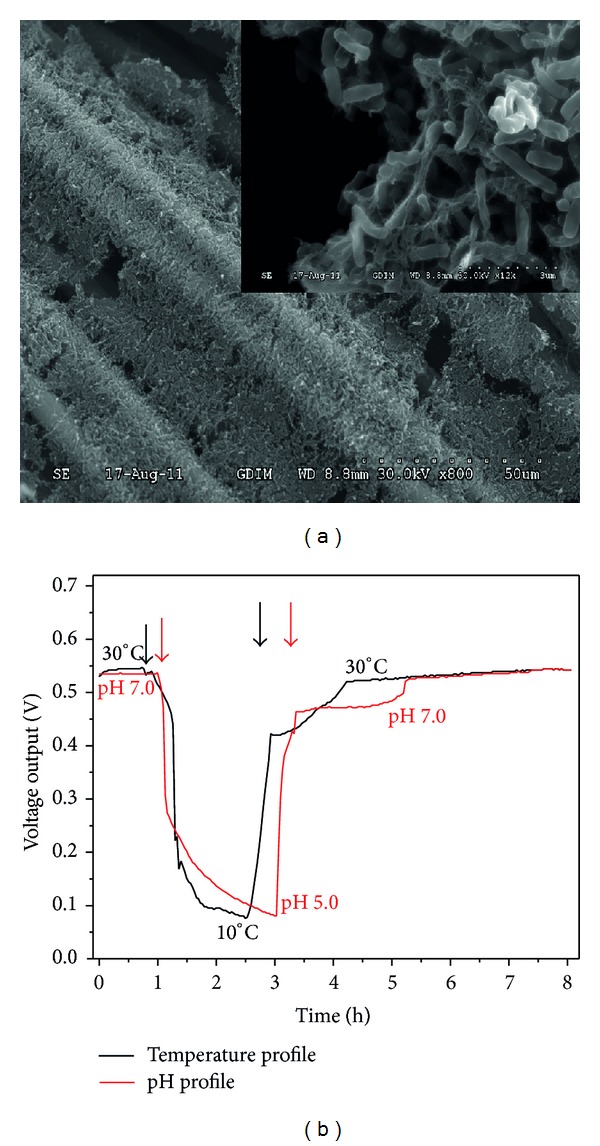
(a) SEM images of the anode biofilm (inset: high resolution image); (b) voltage output versus time curves with the variation of temperature and pH.

An MFC can be a very robust device when it is subjected to short-term changes of operating parameters such as temperature and pH. To probe the response of MFC performance to these parameters, an MFC inoculated with a mixed consortium was constructed. The structure and morphology of the self-assembled mixed-culture bioanode were characterized with SEM (Figure 1(a)). Nearly the entire surface of the solid carbon cloth electrode was covered with rod-shaped bacterial cells. Changes in voltage output in response to temperature and pH changes were shown in Figure 1(b). Voltage output decreased sharply when temperature was lowered from 30°C to 10°C or pH was decreased from 7.0 to 5.0. Previous studies reported similar changes of the voltage output in response to temperature and pH changes [14, 17, 18]. Gonzalez del Campo et al. pointed out that temperature could affect MFC voltage output by influencing microbial metabolism, membrane permeability, and ohmic resistance of the electrolyte [17]. In the case of effects of medium pH on the power generation of an MFC, it was found that acidification of the anode affected electricity generation by inhibiting the microbial activity [18]. In addition, alkaline medium increased biosynthesis of riboflavin from* Shewanella*, which could also enhance bioanode performance [19]. It was important to note here that a rather fast switching speed of voltage output (~0.5 h) was achieved by controlling the pH and temperature, benefiting the next generation of bacteria-based computing circuits or sensors [20].

### 3.2. CVs of Mixed Biofilm as Affected by pH and Temperature

**Figure 2:**
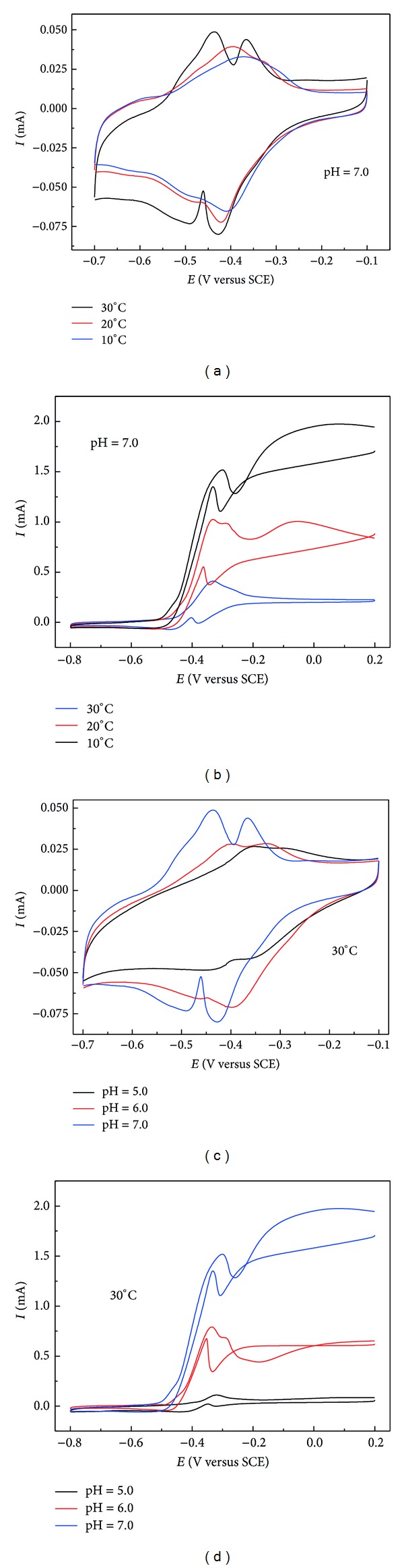
CVs of the anode biofilm under various temperatures by fixing pH condition under no-turnover state (a) and turnover state (b); CVs of the anode biofilm under various pH levels by fixing temperature condition under no-turnover state (c) and turnover state (d).

In addition to the above mentioned reasons, the electrochemical activity of the redox species in the microbial electron transfer chains is also greatly influenced by temperature and pH, further affecting the whole extracellular electron transfer process. Numerous studies claimed that c-type cytochromes (c Cyts) located on the outer membrane of the microbes were the key redox species participating in the extracellular electron transfer [21, 22].  Figure 2 showed the electrochemical properties of the living c Cyts in the mixed-culture biofilm under different temperature and pH conditions, which have been rarely reported in previous studies. As shown in Figure 2(a), similar to the CV features of a pure* Geobacter* strain, there were two pairs of distinct redox peaks on the CV curve for the biofilm in the nonturnover state, with formal potential at −0.46 V and −0.39 V (versus SCE), respectively, at 30°C and pH 7.0. These two couples of redox peaks might belong to two different outer membrane c-type cytochromes of OmcB and OmcZ, respectively [21]. However, the redox peaks positively shifted when the temperature went down. The formal potentials were −0.44 V and −0.37 V (versus SCE) at 20°C and −0.42 V and −0.35 V, respectively (versus SCE), at 10°C. Moreover, the peak separations increased and the peak currents decreased as the temperature decreased, implying decreases in the electrochemical activities of c Cyts. As a result, the electrochemical catalytical activity of the anode biofilm dropped accordingly (Figure 2(b)). It is worth mentioning that temperature caused more significant change in the oxidation peak potential than in the reduction peak potential, demonstrating greater influence of temperature on the oxidation process of the c Cyts. Similar changes in the redox potential of c Cyts were also observed when medium pH was changed. As shown in Figure 2(c), when pH decreased, the redox potentials positively shifted by ca. 40 mV, suggesting that the redox processes of these redox active species were pH-dependent. The peak separations increased and the peak currents decreased as pH decreased, indicating decrease of the electrochemical activity of the c Cyts and the electrochemical catalytical activity of the anode biofilm (Figure 2(d)).

### 3.3. UV-Vis Spectroscopy Measurements of* In Vivo *c Cyts as Affected by pH and Temperature

**Figure 3:**
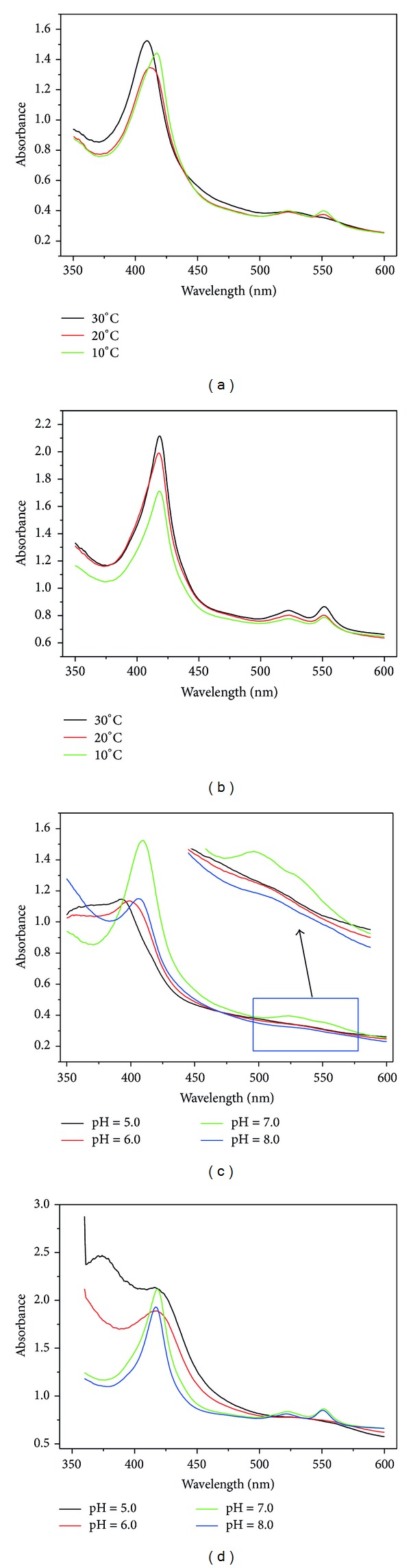
Electronic absorption spectra of* G. sulfurreducens* under various temperatures at pH 7.0 in oxidized state (a) and reduced state (b); electronic absorption spectra under various pH values at 30°C in oxidized state (c) and reduced state (d).

The effects of temperature and pH on* in vivo *c Cyts were further evidenced by investigating the whole cell using a UV-Vis spectroscopy. Bacteria with outer membrane bonded c-type cytochromes showed distinguished UV-Vis spectra with different redox states [23]. The oxidized c Cyts have a Soret band at 409 nm and a broad band at 528 nm (Figure 3). After c Cyts were reduced by sodium dithionite, reduced c Cyts show the Soret, *β*, and *α* bands at 419, 520, and 550 nm, respectively. A similar shift in the Soret absorption band was reported for purified c Cyts [24]. These spectral features are typical of hexacoordinated low-spin hemes and are obviously affected by environmental stimuli. An apparent red shifting of the Soret band and the absence of the peaks at 522 and 552 nm was observed for the oxidized living c Cyts while the temperature went down (Figure 3(a)). However, no shifting but a decrease in intensity of the absorbance peaks for the reduced c Cyts was observed when temperature decreased (Figure 3(b)). The spectral features also changed when the medium pH was adjusted. The Soret band of the oxidized c Cyts shifted to 393 nm with a lower intensity (Figure 3(c)). The position and the intensity of the Soret absorption band were related to the conformational state of the heme group in c Cyts and the weakening of the heme crevice [25]. The appearance of the Soret band at 393 nm demonstrated the formation of a fully high-spin heme complex. No obvious shifts of the Soret band but a weakening in absorption intensity for all the bands were observed when the medium became acid. On the other hand, the blue shifts of the Soret absorption band of both the oxidized and reduced cytochromes were also observed under an alkaline condition. The results suggested that environmental stimuli apparently had an effect on the conformational state of the heme groups in c Cyts and in turn the electrochemical properties of the c Cyts, which provided an opportunity for tuning the extracellular electron transfer of the whole cell.

### 3.4. The Combined Effect of pH and Temperature on the Bioelectricity Generation from the MFC

**Figure 4:**
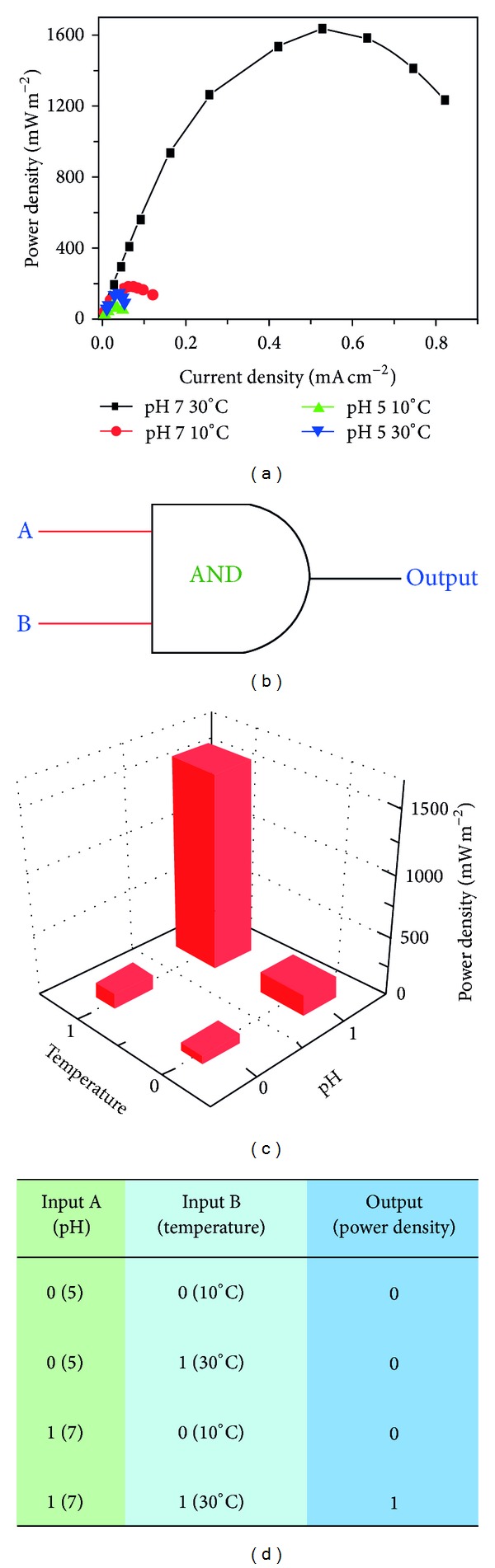
Polarization curves of the MFC under various conditions (a); equivalent circuit of an AND logic gate based on the MFC (b); the maximum power output of the MFC for four different input combinations (c); and truth table for the logically controlled power output (d).

To take advantage of the switchable electrochemical activity of the whole cell by the combined effect of pH and temperature, we constructed a “smart” MFC. In such a device, bioelectricity generation from the MFC could be controlled by temperature and pH. As shown in Figure 4(a), the highest power output was achieved under the conditions of 30°C and pH 7.0, while a very low power output was generated under conditions of lower temperature or pH. Based on this, a Boolean AND logic operation was developed for power generation. In this logic operation, pH and temperature were designed as input signals (A and B signals, resp.), and the maximum power output was taken as output signal (Figure 4(b)). Conditions of pH 7.0 and 30°C were considered as logic input 1, and conditions of pH 5.0 and 10°C were considered as logic input 0. The input signals were applied in all four combinations (0,0; 0,1; 1,0; and 1,1) as shown in Figures 4(c) and 4(d). The highest power density for all MFC tests was obtained for input 1,1 (1600 mW m^−2^). In contrast, the power output was at a low level when input was 0,0, 0,1, or 1,0. The threshold of the Boolean logic AND gate for the MFC was 180 mW m^−2^.

## 4. Conclusion

In this study, the “smart” MFC presented above demonstrated for the first time the possibility to control the power output of an MFC by adjusting environmental conditions according to Boolean logic operations. This provides opportunities for future sensor and bioelectronic devices logically controlled by physiological parameters. “Smart” MFCs can be a promising alternative to enzyme-based biofuel cell systems due to their long-term stability, scalability, and easy handling properties. An MFC with switchable and tunable power release might broaden its application in sensor, medical, and environmental fields.
